# Job Motivation, Burnout and Turnover Intention during the COVID-19 Pandemic: Are There Differences between Female and Male Workers?

**DOI:** 10.3390/healthcare10091662

**Published:** 2022-08-31

**Authors:** Milton Ismael Paredes-Aguirre, Holger Raúl Barriga Medina, Ronald Enrique Campoverde Aguirre, Ester Rebeca Melo Vargas, Mary Betty Armijos Yambay

**Affiliations:** 1 Escuela Superior Politécnica del Litoral, ESPOL, Facultad de Ciencias Sociales y Humanísticas, Campus Gustavo Galindo, Km. 30.5 Vía Perimetral, Guayaquil P.O. Box 09-01-5863, Ecuador; 2 EGADE Business School, Tecnológico de Monterrey, Carlos Lazo 100, Santa Fe 01389, Mexico

**Keywords:** burnout, motivation, turnover intention, gender analysis

## Abstract

The working conditions during COVID-19 highlight the relevance of workers and their occupational health and well-being. The pandemic has caused adverse effects on workers and sharpened social and economic problems, such as a gender gap. In this study, with a multisector sample of 1044 Ecuadorian workers, we present a gender analysis where we evaluate how burnout can mediate the relationship between motivation and workers’ intention to leave their jobs in the COVID-19 context. To test the proposed hypothesis, structural equation model (SEM) was used. In the proposed conceptual model, turnover intention was considered as the dependent variable, the two dimensions of motivation (intrinsic and extrinsic) were the independent variables and burnout was tested as a mediating variable. Consistent with pre-COVID-19 research, our findings confirm the incidence of job motivation on burnout and turnover intention. Additionally, through Sobel’s criteria, we determine that burnout has a mediating effect between job motivation and turnover intention. In terms of gender, we find different results for female and male workers through critical ratios. Our study indicates that female and male workers’ burnout and turnover intentions levels are different when intrinsic motivation is present. In contrast to pre-COVID-19 studies that indicated no gender differences on these variables, we associate these results to gender roles in lockdown conditions during the pandemic.

## 1. Introduction

The conditions of the work environment can significantly influence the performance of workers and, consequently, the results of their employment. Therefore, this topic has been explored by several authors for some years. However, during 2020, interest in occupational health resurfaced due to the radical changes that people had to face in their work environment. Although this situation has persisted over time, the events of the COVID-19 has caused adverse effects on workers and firms.

For instance, in Ecuador, the city of Guayaquil was one of the most affected at the beginning of the SARS–CoV-2 spread in Latin America. The health system did not have enough capacity to help all the people infected; therefore, the health centers collapsed and the number of deaths exceeded 400 citizens daily [1]. Consequently, the authorities decided to implement an obligatory confinement and quarantine for several weeks in all the city. This decision forced firms, public institutions, schools and universities to change their usual face-to-face work situations and begin teleworking.

According to the Ministry of Labor of Ecuador [2,3], at the beginning of the pandemic, more than 208,000 workers adopted remote work. This number reflected a 1890% increase compared to the 14,000 people that used this working method in 2016 [2]. Consequently, remote work continued growing, representing 6% of the employed population (457,000 workers) [3]. During the pandemic, the Ecuadorian government developed several regulations regarding teleworking and economic reactivation [4,5,6], with the objective of maintaining employment conditions after lockdown measures and provide a general framework for applying teleworking during the health emergency declaration.

The pandemic generated new discussions over topics related to the well-being of workers [7,8,9]. Between those there are studies evaluating how the pandemic affected the mental health of employees and employers and, thus, their turnover intention (TI). The study carried out by Bufquin et al. [10] found that active workers experienced psychological problems, as well as drug and alcohol use, more than suspended workers. Additionally, the psychological distress did not only increase the probability of alcohol and drug use but also the TI of the individuals. Finally, they also found that workers, with good or bad mental health, mentioned their desire to change their job during the pandemic. Another study carried out by Mirzaei et al. [11] determined that the turnover intention of health professionals increased because of the job stressors caused by the COVID-19 outbreak. Other work carried out by Tabur and colleagues [9] found that the COVID-19 situation increased the turnover intention, especially among health workers, who were put under enormous emotional pressure.

Similarly, even before the spread of the coronavirus, burnout (BO) was considered one of the psychosocial threats that most affected employees and firms [12,13,14]. There are various studies that examine how burnout affects workers, specifically health workers [12,15,16]. For instance, a pre-COVID-19 study among physicians determined that burnout has effects on quality of care, patient satisfaction, turnover and patient safety [12]. A study by Soto-Rubio et al. [17] find that nurses are normally exposed to psychological and social risks caused by stress, and in the context of a pandemic, those risks increased and, therefore, the authors examined emotional work as a factor that moderates BO. They demonstrate that it is possible to evade the negative impact of BO and job satisfaction if people work on their emotional intelligence by restoring their emotions. They mention that this kind of study and the proposal of intervention programs are necessary in the context of health crisis, such as the pandemic of COVID-19. Another study by Thakre and Kawade [18] found that employees who suffer high levels of work stress faced high burnout and lower well-being.

Another recent study by Barriga et al. [19] on workers in different economic sectors in Guayaquil, Ecuador, analyzes the influence of job and family conflict on BO. They found that the incidence of work–family conflict influences burnout. Because of these outcomes, it is relevant to study BO in a context of remote working, quarantine, and lockdown, because these studies can be used as a guide to improve interventions and the well-being of the people suffering in these kinds of emergencies.

Regardless of the multifaceted impacts of COVID-19 on society, there may be different effects in specific social groups, for example, the effect of working conditions on female and male workers. Compared to other crises where male employment is more affected, the decrease in employment due to social distancing measures caused by COVID-19 affected female workers in sectors that have more women as employees more [13,14]. Many studies during the pandemic have shown different results between female and male workers, considering working conditions (for instance, teleworking) as important factors for their well-being [20,21,22,23,24]. According to UN Women [25], the pandemic can cause challenges for women, including difficulties related to unpaid care, violence, overload of work and job loss. Research by Jang et al. [26] highlights the difference between female and male workers in office homeworking. Their findings showed that, overall, women performed more office work at home than men. Therefore, the studies carried out during the pandemic suggest that the impact of COVID-19 on market labor conditions was greater on female than male workers [27]. Theoretically, it is shown in previous studies [28] that a shock affecting labor market may cause an impact on gender roles and force attitudes to be adapted to the prevailing circumstances. The theory of gender roles asserts that society has different expectations for the behavior of female and male workers, thus shaping the division of labor at home [29] and the gender pay gap [30]. Nevertheless, Collins et al. [31] mention that shocks such as COVID-19, which changed the landscape of work, may not always affect negatively gender roles, as male workers could share household roles with female workers. Therefore, the possible effect remains ambiguous and it is relevant to evaluate whether the relationship between the variables analyzed in this study really change by gender.

In terms of possible outcomes from the implementation of remote work, theories, such as the job demands–resources (JDR), appear relevant [32]. This theory establishes that the conditions at work include demands and resources. Demands related to work involve the environment, the load at work and time pressure, whereas the job resources include recompense, control and support at work and security. Therefore, more job demands can generate more health damage, while an increase in job resources can enable better performance [33]. Moreover, this increase can cause higher levels of motivation and productivity [32,34]. By the other hand, high levels of emotional demands, pressure and a negative perception of the authorities has been associated with turnover intention [35].

In this sense, in this study we evaluate how burnout (BO) can mediate the relationship between motivation on turnover intention (TI) in the COVID-19 context. This work is part of an institutional project, of which a research article has already been published [19], which asses different working environment perspectives of Ecuadorian workers in the COVID-19 context. To conduct the current study, first, we try to uncover the relationship of job motivation on TI. Then, we examine BO as mediator on the relationship between motivation and TI. Additionally, we evaluate the differences that could exist in this relationship depending on the gender. Occupational stress is currently very common and results in complications in workers’ health and the performance of the firm or institution. Several studies have examined the gender differences regarding occupational stress, and while some found that there is no difference between genders, others found that either female workers or male workers suffer more [36]. Some studies have proven that turnover intention, motivation and burnout can manifest differently in male and female workers [37], precisely because of the role of female workers as mothers and also the gender gap at work in terms of salaries and working hours, among other things [25,38].

To develop this study, we carried out an online survey the last week of July 2020. We measured the constructs for this study based on the scales developed by Kuvaas [39] and Dyvsik et al. [40] for intrinsic and extrinsic motivation, respectively; Shirom [41] for burnout; and Cammann et al. [42] for turnover intention. With collected data, we first prove the dimensionality of the scales through confirmatory factor analysis (CFA) using maximum likelihood. After that, we used a structural equation model (SEM) to test the hypotheses. In this case, we also use the maximum likelihood estimation method.

Even though the relationships between job motivation, burnout and turnover intention have been studied separately before [43,44], the originality of this work comes from the fact that we evaluate BO as the mediating variable in the relationship between job motivation and TI during the pandemic. Additionally, we validate the psychometric properties of the scales in a context with few studies on this topic. Finally, we carry out a multi-group and critical ratio analysis to determine whether there are different results between genders, considering the emergency context.

This paper is structured as follows: Section 2 presents a brief theoretical background and hypotheses development. Then in Section 3, we present the methods and how the data were collected and analyzed. Section 4 shows the results of the proposed model. Finally, Section 5 describes the discussion of the results and the conclusion.

## 2. Theoretical Background and Hypotheses Development

The self-determination theory (SDT), which is a job motivation theory, began the discussion of the concepts of intrinsic motivation (IM) and extrinsic motivation (EM). It then developed towards research on work organizations [45]. This theory focuses on the influence of motivation on performance and wellness in organizations. It mentions that if workers have support, and thus motivation, it is not only good for themselves but has collateral positive effects for the organization [46]. According to Deci and Ryan [47], IM is defined as the desire to perform an activity for its own sake or for an individual’s inherent interest and enjoyment. On the contrary, EM is defined as the desire to perform an activity with the intention to attain positive consequences or to avoid negative consequences.

Furthermore, the theory of cognitive evaluation examines the trade-off that could exist between intrinsic and extrinsic rewards (and motivation) [48]. Consequently, recent studies try to analyze this possible trade-off by evaluating how an external motivation, extrinsic reward or punishment can deter the IM of a worker. Regarding this approach, it would be relevant to then see how this interaction ultimately affects the TI of employees [49].

Various studies, such as the ones carried out by Dysvik and Kuvaas [50], Ertas [51] and Panisoara et al. [52], prove the influence of intrinsic and extrinsic motivation on TI. Kuvaas et al. [43] published an empirical study comparing the interaction of both motivations and the impact on TI. They find that IM has a negative influence on TI, whereas for EM, the influence is positive. In the COVID-19 context, Panisoara et al. [52] analyzed the influence of motivation (intrinsic and extrinsic) and burnout on 980 in-service teachers, uncovering the same relationships found as pre-COVID-19 studies.

In terms of gender, aspects that usually motivate people to change jobs include achieving greater career growth or because they have better offers; however, in the case of female workers specifically, turnover intention is often based on no or scarce promotion opportunities and the difficulty of having a balance between family and work [53]. In the case of female workers, they tend to have greater turnover intention because of less motivation due to wage inequality and they do not search for new positions in their organization, but prefer to look for another job [38].

Regarding the complications of motivation because of workload, it is relevant to mention that it is related to turnover intention because female workers find it more difficult to maintain their jobs due to the role society has placed on them, according to the social roles theory. This is a theory of gender based on the stereotypes developed by the different roles that males and females have in society [54]. According to the theory, these roles were defined because of the biological characteristics of men (as hunters) and women (as child-bearers), and even when societies evolved from that prehistoric concept, women continued to have physical demands placed upon them because of their ability to bear children, which has made them less valued in the labor market [55] and they may thus be predisposed towards increased turnover intention. While on the other hand, men have the role of being assets providers, and therefore, their turnover intention tends to be lower, as they are obliged by society to have stable work. Hence, it is relevant to analyze whether the relationship between motivation and turnover intention changes or remains the same when we consider gender.

Therefore, according to what was stated by Kuvaas et al. [43] we hypothesize the following:

**Hypothesis** **H1:** *IM has a negative effect on TI in the COVID-19 context*.

**Hypothesis** **H2:** *EM has a positive effect on TI in the COVID-19 context*.

Furthermore, the mediating role of BO on the relationship between motivation and TI in the COVID-19 context has not been studied previously, but they have been studied separately, utilizing a SDT and JDR approach. Additionally, numerous studies have suggested that not only does BO have an influence on work outcomes directly, but it also has a mediating role between other work-related antecedents and outcomes [45,46,47,48,56,57,58,59].

The concept of burnout (BO) includes a group of various variables, such as affection, cognition, aptitude and attitude, which have a relationship with motivation and the intention to leave [60,61]. According to Shirom et al. [41] BO could be viewed as an effective response to large amount of stress at work, where the job demands surpass the adaptive resources of workers.

The literature studying the relationship between BO and TI has found that they are positively correlated [62,63]. Other research, such as that by Richards et al. [64], shows that BO is a mediator between job stressors and workers’ resulting behavior. Consequently, BO as the result of stress can also be a job stressor that exacerbates psychosocial problems on workers, causing, for instance, turnover [65]. Recent studies, such as those by Back et al. [66] and Sklar et al., [44] proved the mediating effect of BO on TI in the COVID-19 context, using work changes and work-related quality of life as independent variables.

Theoretically and in practice, parenthood is one of the social roles that has its basis in gender. Hence, burnout may affect mothers and fathers differently [67]. Furthermore, female workers who are overemployed tend to experience more conflict at home and may choose to leave their jobs because of exhaustion [68]. Additionally, the importance of gender in the analysis of turnover intention and burnout is relevant because it is well known that most female workers who have a family spend a lot of their time trying to balance family and work, more so than male workers, which can cause them greater stress, hence burnout [37]. Furthermore, this burnout caused by family stress can induce them to leave their job [69]. Similarly, a study by Templeton et al. [70] shows that burnout prevails in female over male workers, and the authors mention that they experience more emotional exhaustion and exhibit more burnout symptoms.

From this, the following hypothesis are derived:

**Hypothesis** **H3:** *BO has a positive effect on TI in the COVID-19 context*.

Moreover, since emotions, social relationships and work satisfaction affect BO [71], IM and EM could have an effect on BO and TI, according to the self-determination theory. For instance, Kim [72] demonstrates, in their study of workers from the Korean public sector, that IM and BO are negatively related, while EM and BO have a positive relationship. In the same way, Lease et al. [73] find that the stress perceived at work is stronger when employees do not experience meaning in and motivation for the work they have.

In terms of gender, in the psychological literature there is a long history of why female and male workers have different educational and occupational motivations. The relevance of this has been changing since the year 2000, when female workers started to obtain higher academic degrees and entering a variety of occupations [74]. Ko and Kim [75] mention that it is important to understand the differences that can exist in motivation and the different possible behaviors between male and female workers that could result into burnout. A study by Memon and Jena [76] mentions that the gender inequality in the workplace is a fact that can significantly affect the motivation and stress of female workers. They mention that the practices of the human resources department should be focused on diminishing this gender inequality and empowering female employees.

Therefore, according to this conceptual context, we derive our fourth and fifth hypotheses:

**Hypothesis** **H4:** *IM has a negative effect on BO in the COVID-19 context*.

**Hypothesis** **H5:** *EM has a positive effect on BO in the COVID-19 context*.

**Figure 1:**
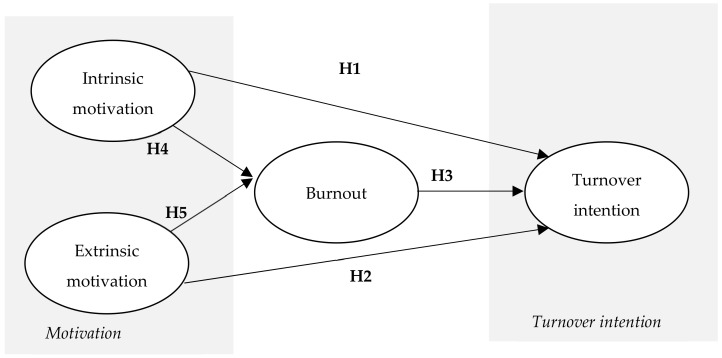
Proposed conceptual model.

From the theoretical framework developed, we summarize in Figure 1 the proposed conceptual model:

## 3. Materials and Methods

### 3.1. Participants and Data Collection

The study design was cross-sectional, and a non-probabilistic convenience sample was used. We collected the data in the second quarter of 2020 using an online survey. Participants were workers from the metropolitan area of Guayaquil (Ecuador) from multiple economic sectors. They were recruited through word-of-mouth and social networks. Participation was voluntary and participants were informed about the importance and objectives of the research and its confidential nature. All participants were asked to indicate that they agreed to participate in the study with an online informed consent form. After data was collected, we performed the analysis of the Mahalanobis distance [77,78,79] to identify the outliers. We finally used a total of 1054 complete and valid records. The respondents were aged between 18 and 79 years old (M = 29.10, SD = 10.02), where 64.64% were millennial workers (from 25 to 42 years). From those, 44.6% (*n* = 470) were male workers, 54.5% (*n* = 574) were female workers and 0.90% (*n* = 10) preferred to not provide information about their gender. Regarding their academic training, most of the sample (43.8%, *n* = 462) had at least a high school diploma, followed by another large group (36.9%, *n* = 389) of professionals with an undergraduate degree. Only 10% (*n* = 105) of the sample were professionals with a Master’s degree and only 8.6% (*n* = 91) and 0.70% (*n* = 7) held specialist technology degrees or a PhD, respectively.

Additionally, on average, the respondents had two (SD = 1.21) people that were economically dependent on them. At the time of data collection, the workers had approximately 12 (SD = 6.58) years of experience, of which 8 (SD = 5.44) were in their current job. The complete sample characteristics are shown in Table 1.

### 3.2. Measurements

The instrument involved the four scales mentioned in the present study: burnout, intrinsic motivation, extrinsic motivation and turnover intention. In this way, these sections have in sum 27 items, to which the participants had to respond according to a 5 point-Likert where 1 represents “totally disagree” and 5 corresponds to “totally agree”.

To measure burnout, we use the 14-item scale developed by Shirom [41]. This scale is made up of three elements: (i) physical fatigue, (ii) cognitive fatigue and (iii) emotional exhaustion. Regarding motivation, we used the intrinsic and extrinsic motivation scales, proposed by Kuvaas [39] and Dysvik [40], respectively. On the one hand, intrinsic motivation consists of 6 items related to the willingness to perform tasks without the need for an external incentive [80]. Whereas the extrinsic motivation is composed of 4 items related to the willingness to perform tasks through the intervention of an external incentive [40]. Finally, we used the 3-item scale of Cammann et al. [42] to measure the turnover intention, which represents the extent to which a person plans to leave their current job.

### 3.3. Data Analysis

We calculated the Kaiser–Meyer–Olkin index and Barlett’s sphericity to evaluate the adequacy of the items and determine the sample adequacy to carry out a confirmatory factor analysis (CFA) [81]. Since, in the conceptual model, there are several relationships between variables, this study used as a statistical technique the structural equation model (SEM). SEM is considered an appropriate method for this analysis since it considers the measurement error in analyzing the variables and their relationships [82]. SEM lends itself well to data analysis for inferential purposes [83]. In this sense, SEM allows testing complex hypotheses about the relationships between observed items and latent variables through the measurement model, and the relationship between latent variables through the structural model [84]. So, we first tested the measurement model using CFA, and secondly, we estimated the structural model to contrast the hypotheses of the proposed conceptual model. We assessed the CFA and the SEM using the maximum likelihood (ML) method, which is based on the covariances of the errors. Additionally, it is considered a robust method for large samples, even with non-normal data distributions [85]. We established a measurement model for each factor, as these were not observable. BO was considered a mediating variable between IM and EM, and the TI has been carried out in previous studies, such as in Ding et al. [86].

We assess the goodness of fit of the scales using global fit index (GFI), chi-square/degrees of freedom ratio (χ^2^/DF), comparative fit index (CFI), Tucker–Lewis index (TLI) and mean square error of approximation (RMSEA). According to the guidelines used, χ^2^/DF must be less than 3, the CFI and TLI values must be greater than 0.95 and the RMSEA values must be less than 0.06 to indicate an acceptable fit with a confidence interval 90% [87].

The factor structure of the latent variables, convergent and discriminant validity were assessed by CFA. According to sample characteristics, we performed the convergent validity analysis by evaluating the internal consistency indicator of Cronbach’s alpha (α), omega (ϖ) and factor loadings [88]. The discriminant analysis was performed using the Fornell and Larcker criterion and cross-loads [89]. We performed those analysis with the packages: Tidyverse, Haven, MBESS, Lavaan, Psych, semPlot, Openxlsx, Ltm, Car, cSEM, semTools and e1071 of the R Studio version 4.0.

## 4. Results

### 4.1. Descriptive Analysis

In Table 2 we show the descriptive statistics of the BO dimensions. We add the scores of the items of each dimension to calculate factor values and divide them by the number of items. Regarding the general sample, the BO dimensions showed a mean between 2 to 3 in physical fatigue (2.54, SD = 0.85, Skew = 0.07, Kurt = 0.01), cognitive fatigue (2.19, SD = 0.91, Skew = 0.13, Kurt = 0.03) and emotional exhaustion (2.23, SD = 0.92, Skew = 0.18, Kurt = 0.01). The values of the other factors are shown in Table 2.

### 4.2. Confirmatory Factor Analysis

According to the sample adequacy and sphericity test results, it was confirmed that it was feasible to use factor analysis for the study data matrix, according to the KMO value close to one (KMO = 0.94) and the significance value Bartlett’s test of 0.000. Therefore, we carried out CFA to prove the dimensionality of the variables considered in this research. We assessed the BO scale using a factor structure of three intercorrelated dimensions, whereas we evaluated the IM, EM and TI scales using a one-dimensional factor structure.

The goodness of fit indices of the BO scale (CFI = 0.948; TLI = 0.936; RMSEA = 0.090 [90% CI: 0.084–0.096]) as well as for the IM scales (CFI = 0.938; TLI = 0.897; RMSEA = 0.148 [90% CI: 0.131–0.165]) and EM scales (CFI = 0.938; TLI = 0.897; RMSEA = 0.148 [90% CI: 0.131–0.165]) and TI (CFI = 1.00; TLI = 1.00; RMSEA = 0.00 [90% CI: 0.00–0.00]) were adequate.

#### 4.2.1. Convergent Validity Analysis

To evaluate the internal consistency, Cronbach’s alpha (α), omega (ϖ) and factor loadings were used, considering 0.65 as a critical value, which is a value commonly accepted in social science research [90]. Hence, we show that the BO, IM, EM and TI have an acceptable internal reliability with internal consistency values greater than 0.854. The correlation coefficients were statistically significant for each of the scales, ranging between 0.71 and 0.92 for the BO scale, between 0.69 and 0.86 for the IM scale, between 0.71 and 0.75 for the EM scale and between 0.78 and 0.93 for the TI scale. All these outcomes are shown in Table 3.

We evaluated the convergent validity of the factors using the average variance extracted AVE, considering 0.50 as critical value [91]. From the results obtained, we verified the convergent validity of the BO, IM, EM and TI; the variance extracted is greater than 0.50.

#### 4.2.2. Discriminant Validity Analysis

Proposed by Hair et al. [92], we used the Fornell and Larcker criterion [93] to show that the AVE from the latent variables, which are related in the structural model, is greater than the correlation with the other factors of the structural model. According to the data obtained, we confirm the discriminant validity of the factors of the model, according to the cross-load and Fornell and Larker criteria [93].

### 4.3. Hypothesis Testing

**Figure 2:**
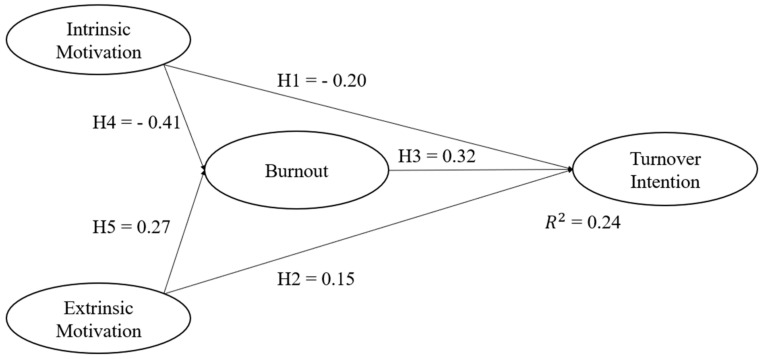
Standardized structural path coefficients. N = 1054.

To prove the hypotheses of the influence of intrinsic and EM on BO and its effect on the TI, we estimated a SEM. We show in the results that IM has a negative and statistically significant effect on BO (β = −0.417, *p* < 0.001) and TI (β = −0.20, *p* < 0.001) and H4 and H1, respectively. Likewise, we show that EM has a positive and significant effect on BO (β = 0.27, *p* < 0.001) and TI (β = 0.15, *p* < 0.001), confirming H5 and H2. Additionally, we found that there is a positive and statistically significant effect with BO and the TI (β = 0.32, *p* < 0.001). Therefore, H3 is proved. We find adequate goodness of fit indices on this specification (CFI = 0.943; GFI = 0.902; NFI = 0.931; TLI = 0.935; RMSEA = 0.065 [90% CI: 0.062–0.069]). Table 4 and Figure 2 present the estimation results.

Additionally, we considered gender to account for variability that could exist when separating male and female workers on the estimation. The SEM specification estimation using male (G1) and female (G2) subsamples showed important differences. In both subsamples, EM has positive and statistically significant effect to BO and TI. The same condition is fulfilled in the relationship between BO and the TI. In contrast, the relationship of IM with BO and TI was negative and statistically significant.

When assessing both subsamples, a stronger effect was found in G2 than in G1 between IM and BO (G2: β = −0.43, *p* < 0.01; G1: β = −0.38, *p* < 0.01) and the TI (G2: β = −0.30, *p* < 0.01; G1: β = −0.06, *p* < 0.01). In contrast, a higher influence was identified in the male subsample than in the female subsample between EM and BO (G1: β = 0.31, *p* < 0.01; G2: β = 0.23, *p* < 0.01). While the association between EM and TI remained the same for both subsamples (G1: β = 0.15, *p* < 0.01; G2: β = 0.15, *p* < 0.01). Finally, the relationship between BO and TI was greater in the G1 subsample (β = 0.40, *p* < 0.01) than in the G2 subsample (β = 0.24, *p* < 0.01).

**Figure 3:**
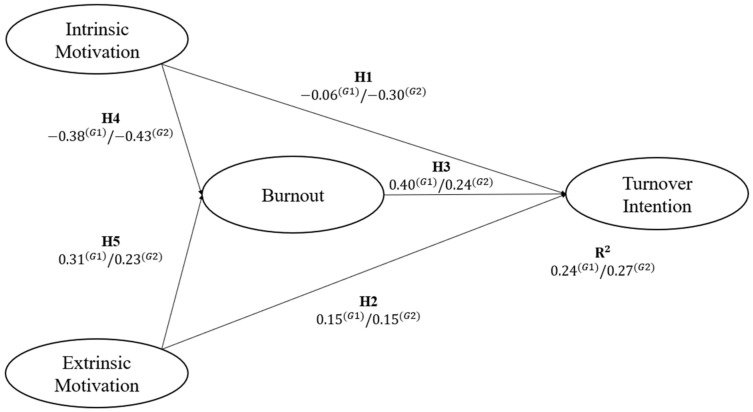
Standardized structural path coefficients for both subsamples. G1: Male (*n* = 470); G2: Female (*n* = 574).

The indices of goodness of fit were CFI = 0.946; GFI = 0.899; NFI = 0.933; TLI = 0.935; RMSEA = 0.073 [90% CI: 0.068–0.078]; and SRMR = 0.053 in the case of the G1; and CFI = 0.927; GFI = 0.847; NFI = 0.901; TLI = 0.912; RMSEA = 0.089 [90% CI: 0.081–0.097]; and 400 SRMR = 0.092 in G2. Therefore, H1, H2, H3, H4 and H5 were confirmed for male and female workers. Table 5 and Figure 3 show the estimates of the trajectory coefficients for both subsamples.

Path coefficients results show that the female subsample (G2) has a behavior similar to that of the general sample, due to the fact that most of the population corresponded to that sex; in contrast to the male subsample (G1). Furthermore, these coefficients were found to be significant according to the *p*-value (*p* < 0.001).

### 4.4. Direct and Indirect Effects

According to Table 6, each increase of one unit in EM was associated with an increase in BO at A = 0.272. Considering the adjusted EM, each increase of one unit in BO was associated with an increase in the TI at B = 0.316. Therefore, the increase in EM was associated with the increase in the TI indirectly through BO. Specifically, for each A = 0.272 increase in EM and BO, there was an AB = 0.086 increase in TI. Finally, the increase in EM was directly associated with the increase in the TI at CP = 0.152.

Contrarily, each increase of one unit in IM relates to a decrease in BO at D = −0.406. Considering the adjusted IM, each increase of one unit in BO was associated with an increase in the TI at E = 0.316. Hence, the increase in IM was associated with the increase in the TI indirectly through BO. Specifically, for each D = −0.406 increase in IM and BO, there was a DE = −0.128 decrease in TI. Finally, the increase in IM was directly associated with the decrease in TI at FP = −0.195.

#### Mediation Analysis

According to Sobel’s criteria [94], we found that the indirect effect of IM on the TI through BO was significant (Sb = −4.456; SE = 0.040; *p* < 0.001). Likewise, the mediating the role of the EM path was also significant (Sb = 6.096; SE = 0.093; *p* < 0.001).

### 4.5. Gender Multi-Group Analysis

By applying multigroup analysis, researchers are able to test for differences between two identical models for different groups [95]. In Table 7, when contrasting the insidence of gender in the model as a whole, the subsamples (male and female) did not show significant differences. This result implies that IM and EM produce the same effect of turnover intention through BO for both the male and female group; therefore, there are no gender differences in this situation.

### 4.6. Critical Ratios for Gender Differences between Parameters

Although the multi-group analysis showed no gender difference, we demonstrated differences between the parameters of the groups (male and female) when quantified through the critical ratios [96] (see Table 8). The relationship between IM and TI presented a difference of −3.449, which means that the male population feels less motivated to leave their job when they satisfy the reasons to be in the office, in contrast to the female population, who do not consider IM as a single and sufficient factor to determine their intention to remain in the job position [97,98]. The relationship between IM and BO presented a difference of −0.749. This result means that female workers have slightly higher job BO than male workers when work does not contribute to satisfying their internal motivations. Finally, the relationship between BO and TI had a difference of −1.794, which means that the female population is more willing to abandon their work when it causes physical, emotional and cognitive exhaustion, unlike the male population, whose extrinsic motivation manages to compensate for their job BO and intention to remain in office [98]. Therefore, it is found significant differences between G1 and G2 in the relationship of IM and its effect on TI through BO.

## 5. Discussion

The present work has compared the influence of motivation and employee TI, considering burnout as the mediating variable in the COVID-19 context. In accordance to pre-pandemic studies [43,72] and recent research [52] our results suggest that IM and EM are potent factors of turnover intention. Intrinsic motivation has a significant negative effect on turnover intention, whereas EM presents a positive significant effect. Additionally, the mediating role of burnout is confirmed through the resultant significant relationship of motivation and burnout, and burnout and turnover intention. Results suggest that the mediation effect of burnout was significative for job motivation. However, findings show more influence of the intrinsic dimension than EM. These outcomes indicate that when a job is satisfying, burnout and turnover intention are reduced.

To identify if gender matters, we separated male and female workers to account for variability that could exist. We find relevant differences when we consider the male and female subsamples for the estimation of the structural equation model. In both subsamples, EM was positive and had a statistically significant relationship with both BO and TI. The same condition is fulfilled in the relationship between BO and the TI. In contrast, we found a significantly negative effect of IM on BO and TI.

When we contrast the model through multi-group analysis, the subsamples of gender did not show significant differences. This result implies that IM and EM produce the same effect of turnover intention through BO for both the male and female group; therefore, there are no gender differences in this situation. However, considering the disparities in standardized estimates we evidenced differences between the parameters of the groups (male and female) when quantified through the critical ratios.

As opposed to some previous studies [43,72], we found gender differences when analyzing job motivation, burnout and turnover intention.

These finding are congruent with previous studies [25,26,37,38,70,75] where female and male workers were affected differently in lockdown circumstances. Recent studies, contrary to the traditional allocation of roles, highlight the fact that many parents in the COVID-19 context have to assume the main responsibility for childcare, which can modify the distribution of labor at home [20]. The differences for female workers and male workers in occupational stress can vary, depending on work demands. Garcia et al. [99] found for example that female workers can experience more stress than male workers. These results are also in agreement with the gender role theory exposed in the theoretical framework. This theory asserts that the society behaves and thinks based on their own beliefs and stereotypes about the roles that females and male workers should have, and certain careers, jobs and attitudes are expected to be from a specific gender [100]. Consequently, female workers are expected to be more sensitive and emotional and are perceived to not be suitable for jobs that demand more effort, which in turn could cause them to suffer more burnout and less motivation [26].

The occupational stress caused by different job demands can be higher for female workers because they have the majority of the responsibilities in the home and in childcare [101]. In the context of COVID-19, the mental health of female workers could have been more affected because of the extra responsibilities at home; during the pandemic, occupational stress increased and gender roles were fundamental to worsening this stress. Because of that fact, a high number of female workers increased their turnover intention and their burnout and decreased their motivation [102].

In our study, the male population feel more predisposed to leave their jobs when intrinsic motivation is present. Contrarily to the female population, who consider IM as an important factor to remain in the job position. Additionally, female workers show slightly higher BO than male workers when work does not contribute to satisfying their internal motivations. Finally, the male population is more willing to abandon their work when it increases their physical, emotional and cognitive exhaustion. Non-significative differences were determined in the extrinsic motivation path. Many factors could explain these different results, but role ambiguity [8] and working from home difficulties during the pandemic [7,19] could highlight previous motivation study results that indicate that social relationships at work and level of achievement can be key motivators for workers, whereas rewards and job characteristics can be secondary [70].

## 6. Contributions

The contribution of this study are focused on three aspects. First, this work is a novel assessment of BO as the mediating variable on the relationship between job motivation and TI. Second, we also performed a validation of the psychometric properties of job motivation and turnover intention scales in the Latin American context. Third, we carried out a multi-group analysis to compare this relationship with gender in a region with traditional norms in the distribution of work and home activities.

Our findings are relevant for managers to alert them to the importance of facilitating adequate working conditions where IM may be promoted to reduce workers’ turnover intentions [43]. It is confirmed that to reduce turnover intention, the levels of burnout should be diminished, and to achieve this, managers may consider job demands and job resources [32,34].

Additionally, our findings can promote the recognition of gender disparity to design policies to eradicate gender inequality during crises, such as the pandemic, for workers with young children, single-parent families and economically vulnerable groups for whom it is more difficult to adapt themselves in health emergency working conditions.

## 7. Limitations

Despite the contributions of this study, there are some limitations that should be mentioned. One of the key limitations was the cross-sectional design. Causal inference changes over time are not possible to determine with cross-sectional data [103]. Future studies should consider longitudinal designs to confirm our findings. Additionally, the data collected were based on self-reports, which can over- or underestimate the observations [104]. Finally, this study focuses on female and male workers. Future studies should focus on this type of study on workers of other genders in order to promote the analysis of the LGBTI + community in the labor market.

## Figures and Tables

**Table 1:** Sample characteristics.

Section		Female		Male	
		Frequency	Percentage (%)	Frequency	Percentage (%)
Age	62–79	4	0.70	14	3.00
	43–61	119	20.70	107	22.80
	25–42	360	62.70	269	57.20
	18–24	91	15.90	80	17.00
	Cumulative %	574	100	470	100
Education	High school and below	216	37.63	243	51.70
	Technical	36	6.27	51	10.85
	Undergraduate	255	44.43	131	27.87
	Graduate	64	11.15	41	8.72
	Doctoral degree	3	0.52	4	0.86
	Cumulative %	574	100	470	100
Dependents at home	0	203	35.37	131	27.87
	1–3	324	56.45	245	52.13
	4–7	45	7.84	92	19.57
	8–11	2	0.35	2	0.43
	Cumulative %	574	100	470	100
Total work experience	0–5	295	51.39	208	44.26
	6–10	99	17.25	84	17.87
	11–20	118	20.56	104	22.13
	21–30	58	10.10	54	11.49
	31–40	3	0.52	19	4.04
	41–50	0	0	1	0.21
	51–60	1	0.17	0	0
	Cumulative %	574	100	470	100
Work experience in current job	0–5	435	75.78	342	72.77
	6–10	77	13.41	63	13.40
	11–20	36	6.27	42	8.94
	21–30	25	4.36	21	4.47
	31–39	1	0.17	2	0.43
	Cumulative %	574	100	470	100
Economic Sector	Agriculture	13	2.26	10	2.13
	Cattle raising	3	0.52	1	0.21
	Aquaculture and Fishing	10	1.74	9	1.91
	Petroleum and Mines	3	0.52	5	1.06
	Industry	63	10.98	68	14.47
	Construction	14	2.44	27	5.74
	Services	254	44.25	208	44.26
	Education	90	15.68	47	10
	Commerce	124	21.60	95	20.21
	Cumulative %	574	100	470	100

N = 1044.

**Table 2:** Descriptive statistics of the latent variables.

Dimensions	Mean	Skewness	Kurtosis	SD
Burnout (BO)				
1. Physical fatigue	2.54	0.07	0.01	0.85
2. Cognitive weariness	2.19	0.13	0.03	0.91
3. Emotional Exhaustion	2.23	0.18	0.01	0.92
Intrinsic motivation (IM)	3.84	−0.13	0.15	0.77
Extrinsic motivation (EM)	3.32	−0.03	−0.10	0.88
Turnover intention (TI)	2.83	0.04	−0.22	1.13

N = 1044.

**Table 3:** Internal consistency and extracted variance.

	Cronbach’s Alpha	Omega	AVE
Burnout (BO)			
1. Physical fatigue	0.906	0.906	0.664
2. Cognitive weariness	0.940	0.940	0.759
3. Emotional Exhaustion	0.883	0.887	0.715
Intrinsic motivation (IM)	0.890	0.890	0.622
Extrinsic motivation (EM)	0.777	0.776	0.548
Turnover intention (TI)	0.916	0.921	0.784

**Table 4:** Standardized path coefficients for the structural model.

Hypothesis	Standardized Coefficient	*p*-Value
H1: IM → TI	−0.20	***
H2: EM → TI	0.15	***
H3: BO → TI	0.32	***
H4: IM → BO	−0.41	***
H5: EM → BO	0.27	***

Note. *** *p* < 0.001.

**Table 5:** Standardized path coefficients for structural model (male and female).

Hypothesis	Standardized Coefficient
Subsample G1: Male	
H1: IM → TI	−0.06 ***
H2: EM → TI	0.15 ***
H3: BO → TI	0.40 ***
H4: IM → BO	−0.38 ***
H5: EM → BO	0.31 ***
Subsample G2: Female	
H1: IM → TI	−0.30 ***
H2: EM → TI	0.15 ***
H3: BO → TI	0.24 ***
H4: IM → BO	−0.43 ***
H5: EM → BO	0.23 ***

Note. *** *p* < 0.001.

**Table 6:** Direct and indirect effects.

	Indirect Effects		Direct Effects		Total Effects	
	TI	BO	TI	BO	TI	BO
BO	-	-	0.316 ***	-	0.316 ***	-
IM	−0.128 ***	-	−0.195 ***	−0.406 ***	−0.323 ***	−0.406 ***
EM	0.086 ***	-	0.152 ***	0.272 ***	0.238 ***	0.272 ***

Note. *** *p* < 0.001.

**Table 7:** Multigroup analyses results for gender.

Model	χ^2^/DF	TLI	CFI	RMSEA (90% CI)	*p*-Value	Δ CFI	Δ RMSEA
M1. Unconstrained	3.595	0.925	0.934	0.050 (0.047–0.052)	***		
M2. Equal factor loadings	3.505	0.928	0.934	0.049 (0.047–0.052)	***	0.000	−0.001
M3. Equal direct effects	3.501	0.928	0.933	0.049 (0.047–0.051)	***	−0.001	−0.001
M4. Equal structural variances/covariances	3.484	0.928	0.933	0.049 (0.046–0.051)	***	−0.001	−0.001
M5. Equal structural residual variances/covariances	3.480	0.928	0.933	0.049 (0.046–0.051)	***	−0.001	−0.001
M6. Equal measurement error variances/covariances	3.421	0.930	0.931	0.048 (0.046–0.051)	***	−0.003	−0.002

χ^2^/DF = chi-squared/degree freedom, TLI = Tucker–Lewis index, CFI = comparative fit index, RMSEA = root mean square error of approximation, *** *p* < 0.001.

**Table 8:** Critical ratios analysis.

Hypothesis	Difference
H1: IM → TI	−3.449 **
H2: EM →TI	0.000
H3: BO→ TI	−1.794 **
H4: IM → BO	−0.749 **
H5: EM → BO	0.000

Note. ** *p* < 0.05.

## Data Availability

The data presented in this study are available on request to the corresponding authors. The data are not publicly available due to privacy concerns.
